# Relative Age Effects in Male Cricket: A Personal Assets Approach to Explain Immediate, Short-Term, and Long-Term Developmental Outcomes

**DOI:** 10.3390/sports10030039

**Published:** 2022-03-05

**Authors:** Adam L. Kelly, Thomas Brown, Rob Reed, Jean Côté, Jennifer Turnnidge

**Affiliations:** 1 Centre for Life and Sport Sciences (CLaSS), Faculty of Health, Education and Life Sciences, Birmingham City University, Birmingham B15 3TN, West Midlands, UK; thomas.brown7@mail.bcu.ac.uk; 2 Warwickshire County Cricket Club, Birmingham B15 3TN, West Midlands, UK; 3 Corsham Cricket Club, Corsham SN13 9EU, Wiltshire, UK; rob@f5design.co.uk; 4 PLAYS Research Group, School of Kinesiology and Health Studies, Queen’s University, Kingston, ON K7L 3N6, Canada; jc46@queensu.ca (J.C.); jm123@queensu.ca (J.T.)

**Keywords:** talent identification, talent development, expertise, youth cricket, batting, bowling

## Abstract

The purpose of this study was to adopt the Personal Assets Framework (PAF) to examine the immediate, short-term, and long-term developmental outcomes associated with relative age effects (RAEs) in male cricket. As such, this study was comprised of three aims: (a) examine the birth quarter (BQ) distribution of players throughout the England and Wales Cricket Board (ECB) national talent pathway (i.e., Regional U15, Regional U17, England U19, England Lions, England T20, England ODI, and England Test; *n* = 1800; immediate timescale), (b) explore the youth-to-senior transitions based on BQ and skill-set (i.e., batters and bowlers; short-term timescale), and (c) analyse the average number of games played at senior levels based on BQ and skill-set (i.e., long-term timescale). A chi-square goodness of fit test, Cramer’s V, odds ratios, and 95% confidence intervals were used to compare the BQ distributions of each cohort against the expected BQ distributions. In the immediate timescale, results showed that relatively older players were overrepresented throughout all the youth levels (*p* < 0.05, V = 0.16–0.30), whereas there were no differences at senior levels (*p* > 0.05, V = 0.05–0.15). In the short-term timescale, when the senior cohorts were compared to the expected BQ distributions based on the Regional U15 cohort, relatively younger players were more likely to transition from youth to senior levels (*p* < 0.05, V = 0.22–0.37). In the long-term timescale, relatively older batters were selected for more games (*p* < 0.05, V = 0.18–0.51), whereas relatively younger bowlers were selected for more games (*p* < 0.05, V = 0.17–0.39). Moving forward, it is important for researchers and practitioners to better understand how (bi)annual-age grouping shapes developmental outcomes in across different timescales (i.e., immediate, short-term, and long-term), as well as consider alternative grouping strategies and RAE solutions.

## 1. Introduction

Identifying athletes with the potential to achieve expertise at adulthood is a contemporary challenge for many national governing bodies in sport [1]. In the context of cricket, the England and Wales Cricket Board (ECB) are tasked with the design, implementation, and evaluation of their respective national talent pathway in order to facilitate the next generation of senior international players. However, the difficulty of accurately predicting future performance abilities can result in biases during the selection process into talent pathways [2,3]. Particular selection and developmental biases that have been consistently highlighted in the literature are relative age effects (RAEs) [4]. Relative age effects illustrate that when athletes are banded according to (bi)annual-age groups, those who are born near the beginning of the cut-off date are often overrepresented in recreational and talent pathways compared to those who are born towards the end [5]. Indeed, it is important that stakeholders employed at the ECB and in youth cricket settings better understand how RAEs may affect their talent pathway, in order to ensure they are using resources most effectively, providing an equitable system, capture a wide pool of potential talent, and better understand how they may impact individual development based on skill-set (i.e., batters and bowlers). Possible explanations that have been offered for RAEs include the enhanced physiological and psychosocial skills of relatively older athletes, which allows them to outperform their relatively younger but age-matched peers [6,7,8,9]. More specifically, if relatively older athletes are selected because of their physiological and psychosocial qualities, they may gain access to more coaching and competition opportunities, which could allow them to become better athletes in the long-term [8,9,10]. In comparison, studies have shown detrimental effects for relatively younger athletes, such as limited selection opportunities, lower participation, and higher dropout rates [11,12].

Despite being vulnerable to RAEs, little research specifically in cricket exists. Indeed, where it does, early evidenced was offered through limited sources. For instance, RAEs in cricket were first studied in a Letter to the Editor [13], which analysed the birth distribution of British male county competition based on their skill-set during the 1990/91 season. They revealed RAEs in fast bowlers but not for spin bowlers, batters, or wicketkeepers. However, it should be noted that their cut-off dates were misinterpreted, since the April 1st to March 31st were used in-line with the cricket season, whereas the annual selection year representative of national normative data (i.e., September 1st to August 31st in the UK) is generally accepted as the appropriate analysis procedure [14]. Thereafter, using such approach, conference proceedings [15,16], as well as unpublished doctoral thesis data [17], found no evidence of RAEs in senior level cricket. Interestingly, however, RAEs across Under (U)12 to U17 English cohorts have been revealed, whereby those born earlier in the annual selection year were overrepresented compared to those who were born later (birth quarter [BQ]1 = 32–44% (i.e., born in September, October, or November) vs. BQ4 = 11–15% (i.e., born in June, July, or August)); although there were no statistically significant RAEs at U19 level (e.g., BQ1 = 29% vs. BQ4 = 20%) [17]. It is important to note that the number of years included in this study varied considerably and only included limited longitudinal data that may not fully capture the longevity of RAEs throughout the ECB talent pathway.

More recently, RAEs were examined in the male and female Australian youth national championships and senior state competition [18]. Results indicated that players born in the first quartile of the cricket season were significantly overrepresented in male U15, U17, and U19 levels, as well as female U15 and U18 levels (BQ1 = 34–38%), compared to the fourth quartile (BQ4 = 16–20%). In contrast, there were no significant differences at the senior state levels for either male or female cricketers. In comparison to senior levels, RAEs were investigated in ‘super-elite’ senior international test cricketers over a 20-year period according to eleven performance criteria [19]. Results revealed RAEs were prevalent when all skill-sets were combined, and was also observed for the batting and spin bowling skill-sets; although no RAEs were found for the pace bowling skill-set. Similarly, it was shown how England senior international spin bowlers were relatively older when compared to their England senior professional counterparts [20]. Moreover, career batting averages of England senior international players were used to create samples of high-performing and low-performing batters [21]. Results showed how ‘high-performers’ were 1.6 times more likely to be born in BQ1 compared to BQ4, whereas there was no significant difference in the BQ distribution for the low-performers. Contrastingly, however, other research has showed how RAEs did not discriminate between English senior international and professional batters [20]. Indeed, this supports other existing cricket research that illustrates how differential RAEs are contingent on batting or bowling skill-sets. Upon examining the youth-to-senior level transition, a ‘reversal effect’ of relative age was reported in the ECB national talent pathway [22]. They showed how relatively older players were significantly overrepresented at youth level (e.g., BQ1 = 36% vs. BQ4 = 16%), whereas a significantly greater proportion of relatively younger players successfully transitioned to senior international levels (e.g., BQ1 = 2.5% vs. BQ4 = 6.7%). Although these preliminary findings serve as a useful opening, they did not consider various competition levels, skill-sets, or performance outcomes at adulthood. Thus, it would be worthwhile further exploring the mechanisms of RAEs in the ECB national talent pathway in order to better understand who is at risk of RAEs during the immediate, short-term, and long-term timescales in cricket.

In an effort to better understand the far-reaching implications of RAEs, this phenomenon can be examined through the lens of youth development. Indeed, grounding relative age research in theory is imperative to advancing this field [23,24]. As an example, an initial model was offered by Hancock and colleagues [9], which focused on the role social agents (i.e., parents, coaches, and athletes) in creating and perpetuating RAEs. Shortly thereafter, Wattie and colleagues [25] suggested a constraints-based model (i.e., individual, environment, and task) to explain RAEs. Although there are several applicable youth development models, this current study puts forward the Personal Assets Framework (PAF [26,27,28]) as a representation of development in sport by drawing from the context of cricket. Based on work in developmental and sport psychology, the PAF suggests that there are three key ‘dynamic elements’ required for sport development to occur, including: (a) personal engagement in activities (i.e., the what), (b) appropriate settings and organisational structures (i.e., the where), and (c) quality social dynamics (i.e., the who). When these elements interact with each other, an *immediate* sporting experience is created that can influence *short-term* (e.g., competence, confidence, connection, and character; ‘the 4Cs’) and *long-term* (e.g., performance, participation, and personal development; ‘the 3Ps’) developmental outcomes [29]. By highlighting the key mechanisms (i.e., the dynamic elements) and desired outcomes (i.e., immediate, short-term, and long-term), the PAF provides a useful framework to summarize the potential implications of RAEs on developmental outcomes in sport.

The purpose of this study was to explore the immediate (e.g., selection at youth levels), short-term (e.g., youth-to-senior transitions), and long-term (e.g., games played at senior levels) developmental outcomes due to RAEs in male cricket based on skill-set (i.e., batters and bowlers) through the lens of the PAF. As such, this study was comprised of three aims, including: (a) examine the BQ distribution of players throughout the ECB national talent pathway (i.e., Regional U15, Regional U17, England U19, England Lions, England T20, England ODI, and England Test; immediate timescale), (b) explore the youth-to-senior transitions based on BQ and skill-set (i.e., batters and bowlers; short-term timescale), and (c) analyse the average number of games played at senior levels based on BQ and skill-set (i.e., long-term timescale).

## 2. Methods

### 2.1. Sample and Procedure

Participants included for this study were selected into the ECB national talent pathway between the years of 1998 to 2020 (*n* = 1800). The duration of years varied based on the data that was available for each cohort. The respective duration of years for each cohort are outlined in the tables in the results section. The full dataset that was available for each cohort was gathered to provide the most accurate possible representation. In-line with the ECB national talent pathway, participants were selected for either: (a) Regional U15 (*n* = 914; a total of 65% of birthdates were publicly available for the Regional U15 cohort), (b) Regional U17 (*n* = 296; a total of 94% of birthdates were publicly available for the Regional U17 cohort), (c) England U19 (*n* = 170), (d) England Lions (*n* = 131), (e) England T20 (*n* = 91), (f) England ODI (*n* = 103), and (g) England Test (*n* = 95). All data was extracted from the public website *Cricket* *Archive* [30]. Aligning with the English annual-age group cut-off dates, this methodology divided the year into four equal BQs, starting with September 1st as month one and ending with August 31st as month twelve [1]. Accordingly, each participant was allocated a BQ that aligned with their birthdate to create an observed BQ distribution within each of the ECB national talent pathway cohorts. The observed BQ distributions from each cohort were subsequently compared against National Norms (i.e., the expected BQ distribution calculated from average national live births; Office for National Statistics (ONS)) [31]. Birth quartiles were adjusted for those participants born outside of the UK and emigrated after the age of 16 years to align with their original schooling system (e.g., Australia, India, New Zealand, South Africa, and Zimbabwe apply January 1st as month one and December 31st as month twelve; *n* = 17).

To examine the likelihood of achieving senior international levels (i.e., England T20, England ODI, and England Test) following entry into the talent pathway at youth level [32], the senior BQ distributions were compared against the Regional U15 BQ distribution (i.e., entry vs. expertise [1]). Further, players within the senior cohorts were allocated into groups based on their skill-set (i.e., batters or bowlers). Batters were defined as players who had batted for the majority (≥75%) of their innings in the top six of the batting order. Bowlers were defined as those who bowled at least one over in the majority (≥75%) of games they played. The specific skill-set distributions included: (a) England T20 (batters *n* = 35; bowlers *n* = 50), (b) England ODI (batters *n* = 33; bowlers *n* = 58), and (c) England Test (batters *n* = 46; bowlers *n* = 43). The number of games played by each participant within the senior cohorts based on their BQ distribution was then collated to analyse career selection at adulthood.

### 2.2. Data Analysis

A chi-square (*χ*^2^) goodness of fit test was used to compare the BQ distributions of each cohort against the expected BQ distributions, following procedures outlined by McHugh [33]. Cramer’s V were used to highlight the magnitude of differences between BQ distributions. Conventional thresholds for the Cramer’s V analysis were applied, whereby a value of 0.06 or more indicated a small effect size, 0.17 or more indicated a medium effect size, and 0.29 or more indicated a large effect size [34]. Odds ratios (ORs) and 95% confidence intervals (CIs) were calculated to compare the likelihood of each BQ being selected. Results were considered significant at *p* < 0.05.

## 3. Results

With regards to the immediate timescale, there was a significant difference between the Regional U15 BQ distribution when compared to the National Norms, with a large effect size (*χ*^2^(df = 3) = 108.18, *p* < 0.001, V = 0.30; see Table 1). Significant ORs identified an increased likelihood of relatively older players being selected, with the highest being BQ1 vs. BQ4 (OR 3.78; CI 2.65–5.40). Similarly, there was a significant difference between the Regional U17 (*χ*^2^(df = 3) = 29.81, *p* < 0.001, V = 0.23) and the England U19 (*χ*^2^(df = 3) = 8.25, *p* < 0.041, V = 0.16) BQ distributions when compared to the National Norms, with medium and small effect sizes, respectively. The ORs identified an increased likelihood of relatively older players being selected, with the highest being BQ1 vs. BQ4 for both U17 (OR 2.50; CI 1.53–4.06) and U19 (OR 1.83; CI 0.99–3.39). In comparison, no senior cohort (i.e., England Lions, England T20, England ODI, and England Test) displayed any significant differences between BQ distributions when compared to the National Norms.

When comparing the senior cohorts BQ distributions with the expected BQ distributions based on the Regional U15 cohort to examine the short-term timescale, there was a significant difference, with medium to large effect sizes, in all cohorts: (a) England Lions (*χ*^2^(df = 3) = 35.02, *p* < 0.001, V = 0.37), (b) England T20 (*χ*^2^(df = 3) = 15.24, *p* = 0.002, V = 0.29), (c) England ODI (*χ*^2^(df = 3) = 10.10, *p* = 0.002, V = 0.22), and (d) England Test (χ^2^(df = 3) = 19.36, *p* < 0.001, V = 0.32). Significant ORs identified an increased likelihood of relatively younger players to transition to the senior cohorts, with the highest being BQ4 vs. BQ1 in England Lions (OR 3.9, CI 1.81–8.42), England T20 (OR 3.1, CI 1.21–7.41), England ODI (OR 2.5, CI 1.01–5.97), and England Test (OR 3.6, CI 1.43–9.15).

When analysing the average number of games played at senior levels (i.e., England T20, England ODI, and England Test) to explore the long-term-timescale, each BQ distribution displayed a significant difference when compared to the National Norms, with small to medium effect sizes, favouring BQ1 in England T20 (*χ*^2^(df = 3) = 17.37, *p* < 0.001, V = 0.08), and BQ2 in both England ODI (*χ*^2^(df = 3) = 169.27, *p* < 0.001, V = 0.14) and England Test (*χ*^2^(df = 3) = 289.43, *p* < 0.001, V = 0.25; see Table 2). With regards to skill-sets and games played, batters across the senior cohorts displayed a significant difference in their BQ distributions when compared to the National Norms, with medium to large effect sizes, favouring those who are relatively older: (a) England T20 (*χ*^2^(df = 3) = 51.12, *p* < 0.001, V = 0.18), (b) England ODI (*χ*^2^(df = 3) = 566.56, *p* < 0.001, V = 0.39), and (c) England Test (*χ*^2^(df = 3) = 663.81, *p* < 0.001, V = 0.51). Significant ORs identified an increased likelihood of relatively older batters playing more games at senior levels, with the largest being BQ1 vs. BQ4 in England T20 (1.9, CI 1.40–2.46), and BQ2 vs. BQ4 in both England ODI (OR 6.9, CI 5.42–8.78) and England Test (OR 10.3, CI 7.62–13.82). In contrast, bowlers across the senior cohorts displayed a significant difference in their BQ distributions when compared to the National Norms, with large effect sizes, favouring those who are relatively younger: (a) England T20 (*χ*^2^(df = 3) = 40.31, *p* < 0.001, V = 0.17), (b) England ODI (*χ*^2^(df = 3) = 352.23, *p* < 0.001, V = 0.29), and (c) England Test (*χ*^2^(df = 3) = 292.27, *p* < 0.001, V = 0.39). Significant ORs identified an increased likelihood of relatively younger bowlers playing more games at the senior levels, with the largest being BQ3 vs. BQ1 in both England T20 (1.9, CI 1.42–1.57) and England ODI (OR 3.7, CI 2.05–4.47), and BQ4 vs. BQ1 in England Test (OR 4.3, CI 3.29–5.73).

## 4. Discussion

The purpose of this study was to explore the RAEs throughout the ECB national talent pathway through the lens of the PAF. In the immediate timescale, key findings revealed there was a systematic selection bias throughout all the youth levels (i.e., Regional U15, Regional U17, and England U19), whereby relatively older players were significantly overrepresented when compared to their relatively younger peers. At senior levels (i.e., England Lions, England T20, England ODI, and England Test), however, there were no significant differences between BQ distributions. When the senior cohorts were compared to the expected BQ distributions based on the Regional U15 cohort to explore the short-term timescale, relatively younger players were significantly more likely to transition from youth to senior levels when compared to their relatively older counterparts. With regards to the long-term timescale, based on skill-sets, batters and bowlers displayed contrasting BQ distributions at the senior levels, whereby relatively older batters were selected for significantly more games, whereas relatively younger bowlers were selected for significantly more games. In an attempt to better understand the immediate, short-term, and long-term implications of RAEs within the ECB national talent pathway, this discussion contextualises these key results using the PAF.

### 4.1. Immediate Timescale: How Sport Experiences Are Shaped

When the three dynamic elements of the PAF (i.e., personal engagement in activities, appropriate settings and organizational structures, and quality social dynamics) interact with one another, an *immediate* sporting experience is created. This has a subsequent impact on developmental opportunities in youth cricket, and thus can help explain how RAEs occur during the immediate timescale.

### 4.2. Personal Engagement in Activities

Selection into a talent pathway in cricket can offer a range of immediate benefits, such as gaining access to quality coaching, facilities, higher competition levels, and holistic development opportunities [35]. If those young cricketers who are relatively older are provided with greater openings into talent pathways due to their age, they are inevitably going to be exposed to more fruitful training and developmental activities. In contrast, those who are not selected into talent pathways due to being relatively younger will lose out on engaging in such activities. Moreover, fun, enjoyment, and interest may be heightened for relatively older players due to outperforming and/or being perceived as superior compared to their relatively younger peers. Indeed, this could have a positive impact on immediate cricket experiences at young ages, which may explain the greater proportion of relatively older players at youth levels in this current study. It is also important to realise that RAEs are prevalent from early childhood in sport [5] and are amplified in environments that favour practice over play, specialisation over sampling, and early selection based on immediate performance over long-term potential [24]. Many developmental programmes in cricket have established systems that encourage earlier age-specialisation. As an example, selection into county cricket talent pathways in England often takes place as young as aged 9 years [36]. Interestingly, however, this approach has been widely associated with significant pitfalls. Specifically, questions remain over the lack of evidence to accurately predict future performance capabilities at adulthood based on early selection and performance [3]. For instance, following analysis of match performance data at every age group throughout a First-class County’s Cricket pathway (U10 to U19) Brown and colleagues [37] showed how those bowlers who achieved professional status could not be differentiated from their released peers until U17. Moreover, possible drawbacks of engaging in specialised environments (e.g., burnout, injury, and overtraining) have also been associated with selection at young ages [38]. Thus, it is worthwhile exploring the existing organisational structures in order to create more appropriate settings and equitable opportunities for all BQs, as well as help moderate the RAEs shown in this study.

### 4.3. Appropriate Settings and Organisational Structures

The current results show how ORs and effect sizes of RAEs were dependent on competition level, which corresponds with the Australian context [18]. Specifically, RAEs were more pronounced at the youngest (bi)annual-age group (i.e., Regional U15) and slowly declined as age increased until it levelled out at adulthood (i.e., senior cohorts). As an example, BQ1s were 3.8, 2.5, and 1.8 times more likely to be selected when compared to BQ4s at U15 Regional (large effect size), U17 Regional (medium effect size), and England U19 (small effect size) youth levels, respectively. Evidently, the (bi)annual-age grouping policies may contribute to the presence of RAEs throughout the ECB national talent pathway. However, it is important to understand that RAEs are not naturally occurring phenomenon. Rather, they are created by social agents through their decisions, actions, and policies. Organisational structures in cricket can choose to adapt policies to meet the needs of those who participate to create more equitable competition and moderate RAEs. More specifically, they can change how young players are recruited, how competition is structured, and how they interact with players, coaches, parents, communities, and the environments where we engage in cricket [29].

Similar to other possible discriminatory factors such as ethnicity, gender, and religion, the ECB lists *age* as a protected characteristic in their *Anti-Discrimination Code* [39]. Therefore, it is important that organisational structures in cricket attempt to create the most appropriate settings for every young player in order for them to achieve their full potential [14]. Moreover, Jakobsson and colleagues [40] suggest another possible issue of selecting based on (bi)annual-age grouping is the violation of the guiding principles by the United Nations *Convention on the Rights of the Child* (CRC) [41], which was ratified by the UK in 1991 and is referred to in the UK courts in relation to the *Human Rights Act 1998* [42]. Here, *Article 3* in the CRC states how all decisions regarding a child should be made in the best interest of the child. Thus, not only are there possible discriminatory issues surrounding (bi)annual-age selection to consider, but there could also be potential lawful implications too. This leads us to perhaps the most important question: if we cannot make these changes to moderate RAEs in cricket (and sport in general) now, then when?

Since there appears to be pronounced RAEs throughout the ECB national talent pathway, it is important to consider possible relative age solutions and offer directions for future research. Indeed, a range of potential solutions have been proposed in previous studies, such as coach education [43], an age-ordered shirt numbering system [44], avoiding early deselection [45], a selection quota [46], and delaying the selection process [47]. Moreover, literature on alternative grouping strategies to moderate RAEs is limited when compared to the body of research demonstrating its prevalence. Where proposed grouping strategies have been suggested, little evidence has documented their effectiveness or directly implement those [48]. As an example, Kelly and colleagues [14] conceptualised a *flexible chronological approach*, whereby early birth quartiles (i.e., BQ1s) and late birth quartiles (i.e., BQ4s) should be offered the opportunity to ‘play-up’ [49,50] and ‘play-down’ annual-age groups, respectively. Moreover, Kelly and colleagues [51] introduced birthday-banding, where young athletes move up to their next birthdate group on their birthday with aim is to remove particular selection time-points and specific chronological age groups. Other useful strategies that may utiliased in cricket could be drawn from organisational policies incorporated in youth American football (e.g., *age and anthropometric banding* [52,53]) and youth soccer (e.g., *bio-banding* [54,55]). Despite these banding approaches yet to prove their value in reducing RAEs, both strategies appear to systematically address one of the key mechanisms of RAEs, whereby relatively older athletes may have an advanced physiological skill [6]. As such, future research is required to explore the practical implications of these relative age solutions within a youth cricket context.

### 4.4. Quality Social Dynamics

Parents, coaches, and athletes (i.e., social agents) can amplify or mitigate RAEs in youth sports [56]. The Social Agents Model highlights the processes by which social agents influence RAEs in youth sports through three theoretical principles. First, the *Matthew effect* [57], describes how individuals who are initially advantaged are provided with the means to continue their development and further their advantage, whereas those who are initially disadvantaged remain so. In the context of the current findings, batters who are introduced to the game earlier have time to develop complex technical skills that are associated with expertise in batting [58]. Subsequently, these cricketers are most likely to be selected for early talent development programmes. This could also explain the overrepresentation of batters from BQ1 and BQ2 within the senior cohorts. The second and third theoretical principles of the Social Agents Model are the *Pygmalion* [59] and *Galatea* [60] effects. Both these principles refer to the association between the initial expected outcomes and the observed results. However, these expectations differ, whereby the Pygmalion effect refers to external, whereas the Galatea effect refers to internal. With regards to the results of this study, the beliefs and actions of social agents towards successful cricketers are more likely to be positive when compared to those who are less successful [61]. This may further explain why there is an overrepresentation of BQ1 and BQ2s within the youth cohorts when compared to BQ3 and BQ4s.

In order to understand the impact of RAEs on social dynamics, it is important to recognise how these are directly influenced by the rules and regulations of organised youth cricket. For instance, literature examining mixed-age and play can be drawn upon, where evidence exists to suggest that older and younger players can draw unique benefits from playing with each other [51]. On one hand, relatively older players can experience opportunities for leadership and helping of younger peers [62,63]. On the other hand, relatively younger players may benefit from the opportunity to hone their skills and compete against older teammates [14,64]. However, since fixed (bi)annual-age grouping does not allow players to shift between BQs, they will not face a diverse range of experiences or gain the developmental opportunities offered through mixed-age play. Another potential drawback of fixed (bi)annual-age grouping is that it limits different types of social comparison environments. According to Wood and Wilson [65], social comparison theory suggests that athletes rely on peers as a frame of reference to compare themselves, which is used to build self-perceptions such as competence and identity. By limiting opportunities for social comparison due to remaining the same BQ throughout their respective youth trajectories, cricket players face the prospect of a linear pathway and a restricted resilient sense of self throughout development. Given the limited body of literature that has researched the impact of RAEs on mixed-age play, social comparisons, and leadership opportunities, further research is warranted to substantiate these suggestions in the context of youth cricket.

### 4.5. Short-Term Timescale: The 4Cs

In the short-term, relatively older players gradually engage an increased number of practice and competition opportunities organised youth cricket compared to relatively younger players, which may lead to more cricket-specific *competence*. Thus, in order to compete against relatively older batters and bowlers to gain entry into the ECB national talent pathway, relatively younger batters and bowlers may need to develop higher levels of cricket-specific competence. From a seasonal perspective, relatively older players may score more runs and take more wickets, as well as win more matches and league titles due to higher levels of competence (i.e., greater performance outcomes), which may lead to higher levels of *confidence* [66]. In the context of youth soccer, for instance, Augste and Lames [67] revealed that relatively older youth teams achieved higher league rankings in Germany, while Verbeek and colleagues [68] showed how relatively older youth teams accrued more points-per-game in Holland. In relation to youth cricket, if relatively older players are being selected due to their superior (bi)annual-age group performances in order to gain a competitive advantage (i.e., selection focused on winning rather than nurturing future senior players), it could result in limited selection opportunities, lower participation levels, and higher dropout for relatively younger players in the short-term [11,12]. Indeed, it has been suggested that cricketers who adapt quickest to the increased skill and psychological demands of transitioning to higher levels of performance, could be earmarked as being high potential by cricket officials, and transition across the talent pathway sooner as a result [69]. This could partly explain the RAEs that are present at youth levels in the ECB national talent pathway.

Selection and deselection are often conducted on a seasonal basis in cricket in which reveals consistent RAEs, with early success at each new level valued by coaches [69]. Since relatively older players may be perceived as more competent and are overrepresented in the ECB national talent pathway at youth levels, relatively older players could subsequently gain more time with coaches. Here, the coach-athlete *connection* may be influenced by current competence rather than potential [70]. Moreover, higher levels of athletic competence in relatively older players may lead to higher levels of peer acceptance [71]. In the context of the ECB national talent pathway, relatively older players may be unintentionally exposed to greater opportunities to foster positive connections with key stakeholders, allowing them to thrive in talent pathways compared to their relatively younger peers. In contrast, relatively younger players may be presented with greater challenge and are thus more likely to develop higher levels of resilience [22]. It is possible that this may help foster features of *character* that are required during the transition from youth player to established senior player. However, given the lack of evidence in this area, it offers a range of future research directions to examine the association between age and character-related constructs (e.g., social identity, moral engagement, and pro-social behaviours). Although character is often used as a youth selection criteria in many professional cricket clubs (e.g., [72,73]), this may be personified based on a players birth quartile. As a result, the potential pool of talent believed to have the ‘right’ character may be limited, while possible inaccurate decisions due to the subjective nature of what the ‘right’ character actually is could be of concern. It is also important to recognise that there are diverse characters that engage in various activities differently. For instance, selection criteria are often based on traditional factors (e.g., technical skills and physical performance), which could favour relatively older players [6]. In comparison, play-based metrics (e.g., creativity and interest) may help broaden coaches and practitioners’ perceptions of an athlete’s potential rather than focussing on performance-based characteristics [24]. Moving forward, further research is encouraged to help better understand how RAEs can influence character development and how this can differentiate between BQs.

When examining the youth-to-senior level transition, it was revealed that BQ4s were 3.9, 3.1, 2.5, and 3.6 times more likely to successfully transition to England Lions, England T20, England ODI, and England Test compared to BQ1s based on the Regional U15s BQ distribution, respectively. These findings resonate with [22] results, who found a greater proportion of relatively younger cricketers successfully transitioned from youth to senior international levels compared to their relatively older peers. These results may be due to *reversal effects* of relative age, which is a psychologically based explanation of greater ‘growth’ that relatively younger players experience, whereby they are initially disadvantaged during their development due to additional challenges they face [22]. Although RAEs may benefit a greater proportion of relatively younger players in the short-term during the youth-to-senior level transition, little is known about the long-term impact of reversal effects and how they may genuinely influence senior career performance and longevity.

### 4.6. Long-Term Timescale: The 3Ps

When considering skill-set and number of games played at senior level, relatively younger bowlers played more games compared to their relatively older teammates. These findings are contrast with Jones and colleagues [19] who found RAEs in senior international ‘super-elite’ spin bowlers favouring relatively older players, as well as no RAEs for pace bowlers. Gibbs and colleagues [64] put forward the *underdog hypothesis* to explain why more relatively younger senior players may be outperforming their relatively older equivalents. In the context of the current study, relatively younger bowlers may be benefitting from more competitive play against relatively older counterparts throughout their development. However, it is important to consider how to create a ‘BQ4 effect’ for *all* bowlers who may require such challenges during their development to facilitate long-term *performance* outcomes [14].

Interestingly, however, when analysing the number of games played for batters, relatively older players appear to have played more games compared to their relatively younger teammates. For instance, BQ2 batters were 6.9 and 10.3 times more likely to play an England ODI and England Test match when compared to BQ4 batters, respectively. *Long-lasting effects* of relative age have been found across numerous sports at senior international levels [74]. For instance, Lupo and colleagues [75] showed how athletes who were born at the beginning of the selection year were 1.57, 1.34, 2.69, 1.48, and 1.45 times more likely to reach the senior first and second divisions in Italian basketball, rugby union, soccer, volleyball, and water polo, respectively, when compared to those born at the end of year. In the context of cricket, these findings correspond with previous results [21], which also showed how ‘high-performing’ international batters born in BQ1 were significantly more likely to be selected at senior levels when compared to BQ4s. These long-lasting effects may be due to the early selection of relatively older players, who subsequently have greater access to facilities (e.g., access to bowling machines and specialist equipment) and coaches (e.g., creating connections with key stakeholders), to facilitate their long-term performance capabilities. Further, early selected batters are likely to accumulate more hours of random and varied batting-specific practice through adolescence, which [20] highlighted as a key discriminator in achieving ‘super elite’ status. On the other hand, since relatively younger players may not have been offered these same developmental opportunities, they may not have been able to achieve their full potential. Indeed, this also has a knock-on effect by creating a smaller pool of talent to select from at senior levels due to relatively younger players with potential to excel at adulthood being overlooked at younger ages. Possible explanations for the performance differences between skill-sets could lie in the greater physiological requirements for bowlers that may not be achieved until post-adolescence [76], thus making them less exposed to RAEs as relatively younger players have the opportunity to ‘catch-up’. In contrast, batters may require a larger accumulation of practice to develop the necessary perceptual-cognitive skills required to excel at senior levels [77], and therefore making them more vulnerable to RAEs as relatively younger players are less likely to ‘catch-up’. Overall, stakeholders employed in cricket settings should be cautious of the diverse trajectories as well as the long-term development and performance outcomes between batters and bowlers.

The necessity of examining RAEs at more than one point in time is something that was recently encouraged by Schorer and colleagues [78]. This suggestion is reinforced by the limited evidence that has explored the implications of RAEs on the long-term *participation* of relatively younger players. Moreover, despite relatively older players being more likely to be recruited into talent development pathways at youth levels, it also seems they comprise a greater quantity of players who are unsuccessful in achieving senior levels. Thus, although relatively younger players have been reported to drop out of youth sport at young ages due to RAEs [11], it may be suggested this is being replicated by relatively older players at the latter stages of development during the youth-to-senior level transition. Thus, it is important to create immediate sport experiences that foster rich developmental outcomes that help retain players in the long-term independent of their BQ. In addition to possible deselection and dropout, relatively older players are also exposed to possible injury and burnout due to early selection procedures [35]. For instance, McGrath and Finch [79] identified that fast bowlers are most likely to suffer from overuse injuries, such as stress fractures in the lower lumbar spine. Since injuries are more likely to occur during the adolescence growth spurt [80], it is plausible that those who are selected onto talent pathways from an early age are at an increased risk of developing such injuries due to high training loads and subsequently drop out at the latter stages of the talent pathways. Thus, it is important that organisational structures focus on long-term participation strategies when recruiting, developing, and deselecting young cricketers in their talent pathways, in order to avoid dropout, injury, burnout, and promote continued engagement in cricket. Further research is warranted to better understand the long-term implications of RAEs on participation in cricket and help substantiate these suggestions.

When compared to the possible performance and participation implications, little is known about the *personal development* outcomes due to RAEs. Obvious personal benefits for those who attain senior levels includes membership to the Professional Cricketers’ Association (PCA), global tours, heightened media profile, and possibly higher monetary opportunities. Moreover, selected players may gain access to additional support throughout their development (e.g., psychology profiling, nutrition programming, and strength and conditioning), which may positively impact their abilities for adaptive and positive behaviour [81]. Indeed, it is surprising how RAEs can have such a direct impact on whether an individual gains these personal benefits and additional support that can positively shape their adult livelihood. Although the current evidence-base is scarce, these effects can also play an important role in developing psychosocial skills that characterise oneself [9]. Further quantitative (e.g., observational coding, questionnaires) and qualitative (e.g., content analysis, composite narratives) enquiry is needed to investigate RAEs on a broader range of psychosocial mechanisms, such as global wellbeing, leadership skills, moral disengagement, social behaviour, and social identity.

## 5. Limitations

There are important methodological and contextual limitations to consider when interpreting these current results. From a methodological perspective, the data available for the U15 Regional and U17 Regional groups did not provide an entire representation for these particular cohorts. Nevertheless, both these samples offer a large enough representation to draw valid conclusions from and should not be overlooked. Moreover, online data entry began at various different time points for each group, thus the number of years included in each cohort varied. However, we included all the data that was available and captured the longevity of RAEs throughout the ECB talent pathway by including a minimum of seven years within each cohort. In addition, the number of games played that was used as a proxy for performance may not provide an entire reflection of how well a player has consistently played, although it is plausible to suggest that continued selection is an important contributing factor towards success at adulthood.

From a contextual viewpoint, it was the initial aim of this study to capture both male and female pathways. However, in light of the lack of data available within the female pathway, we decided there was not sufficient evidence to include this population. This may be due to the fact that the first professional female cricket league has only recently started in England (i.e., 2020), thus the absence of data available is likely due to the female pathway being far less developed when compared to their male equivalents. As such, it is important to recognise the many relative age lessons learnt in the male pathway from this current study when designing the emerging female structures to ensure the same issues are not recreated, and instead use this as an opportunity to create contemporary organisational structures and more appropriate youth cricket settings [46]. Moreover, it is also important to consider other possible selection and development biases that are prevalent throughout the ECB talent pathways, such as ethnicity [82], relative access to wealth [83], and birthplace effects [84], that may create a recipe to exacerbate some of the existing RAEs [24,25]. Thus, further research adopting a multidimensional approach to explore these obstacles together is required.

## 6. Conclusions

**Figure 1:**
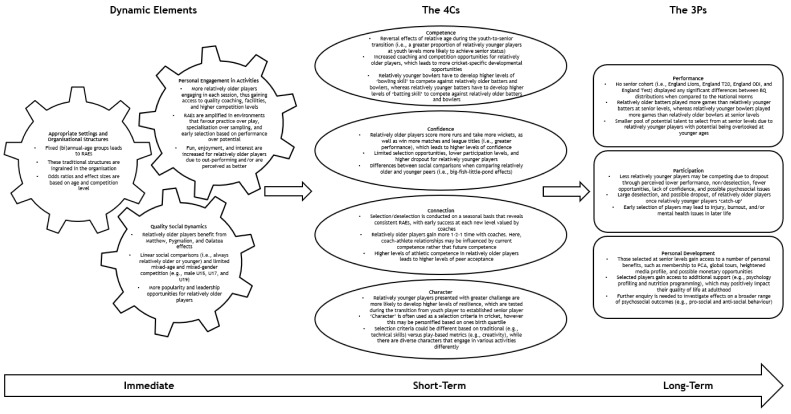
The Personal Assets Framework [26,27,28] as a representation of development in male cricket due to relative age effects.

There appears to be a complex relationship between the month a batter or bowler is born, the likelihood they are selected into a talent pathway, and their opportunities to successfully transition and compete at senior levels. Key results showed how RAEs were prevalent throughout all youth levels (i.e., Regional U15, Regional U17, and England U19), but not at senior levels (i.e., England Lions, England T20, England ODI, and England Test) during the immediate timescale. Moreover, when compared to the expected BQ distribution based on the Regional U15 cohort, BQ4s were between 2.5 to 3.9 times more likely to transition to senior levels when compared to BQ1s during the short-term timescale. In addition, when considering the number of games played at senior levels during the long-term timescale, relatively older batters were selected for more games, whereas relatively younger bowlers were selected for more games. We used the PAF to capture the possible immediate (i.e., dynamic elements), short-term (i.e., the 4Cs), and long-term (i.e., the 3Ps) timescales that are influenced by RAEs based on skill-set (see Figure 1).

Moving forward, it is important for researchers and practitioners to better understand how (bi)annual-age grouping shapes developmental outcomes in sport across different timescales (i.e., the PAF), as well as examine alternative group banding strategies (e.g., a flexible chronological approach, birthday-banding) and possible RAE solutions (e.g., age-ordered shirt numbering, monitoring growth and maturation status). By doing so, it may help create a more appropriate learning environment for every individual to achieve their potential.

## Figures and Tables

**Table 1:** The observed and expected BQ distributions of the ECB talent pathway and senior international cohorts.

Cohort (Percentage Representation)	BQ1 (25.46%)	BQ2 (24.47%)	BQ3 (24.65%)	BQ4 (25.42%)	Total (*n*)	*χ*^2^ (df = 3)	*p*	Cramer’s V	BQ1 vs. BQ4 OR (CI)	BQ4 vs. BQ1 OR (CI)
Regional U15 (2000–2018)	242 (40.5%)	162 (27.1%)	130 (21.7%)	64 (10.7%)	598	108.18	**<0.001**	0.30	3.78 (2.7–5.4)	-
Regional U17 (2013–2019)	105 (37.9%)	72 (25.9%)	59 (21.2%)	42 (15.1%)	278	29.81	**<0.001**	0.23	2.50 (1.5–4.1)	-
England U19 (1998–2020)	55 (32.4%)	47 (27.6%)	38 (22.4%)	30 (17.6%)	170	8.25	**0.041**	0.16	1.83 (1–3.4)	-
England Lions (2011–2020)	31 (23.8%)	31 (23.8%)	36 (27.7%)	32 (24.6%)	130	0.68	0.877	0.05	—	-
England T20 (2005–2020)	23 (25.3%)	23 (25.3%)	26 (28.6%)	19 (20.9%)	91	1.13	0.769	0.08	—	-
England ODI (2000–2020)	28 (27.2%)	32 (31.1%)	25 (24.3%)	18 (17.5%)	103	4.61	0.202	0.15	—	-
England Test (2000–2020)	21 (22.1%)	27 (28.4%)	27 (28.4%)	20 (21.1%)	95	2.11	0.549	0.11	—	-
England Lions (Expected distribution *)	31 (53)	31 (35)	36 (28)	32 (14)	130	35.02	**<0.001**	0.37	—	3.91 (1.9–8.4)
England T20 (Expected distribution *)	23 (37)	23 (25)	26 (19)	19 (10)	91	15.24	**0.002**	0.29	—	3.05 (1.2–7.9)
England ODI (Expected distribution *)	28 (42)	32 (28)	25 (22)	18 (11)	103	10.10	**0.002**	0.22	—	2.45 (1–5.9)
England Test (Expected distribution *)	21 (38)	27 (26)	27 (21)	20 (10)	95	19.36	**<0.001**	0.32	—	3.62 (1.4–9.1)

* Expected distribution calculated from Regional U15 BQ distribution. Bold font denotes statistically significant chi-square at *p* < 0.05.

**Table 2:** The observed BQ distributions of ECB senior international cohorts based on number of games played and skill-set.

Cohort (Percentage Representation)	BQ1 (25.46%)	BQ2 (24.47%)	BQ3 (24.65%)	BQ4 (25.42%)	Total	*χ*^2^ (df = 3)	*p*	Cramer’s V	BQ1 vs. BQ4 OR (CI)	BQ4 vs. BQ1 OR (CI)
England T20	391 (28.25%)	349 (25.22%)	357 (25.79%)	287 (20.74%)	1384	17.37	**<0.001**	0.08	1.36 (1.1–1.7)	—
England ODI	926 (21.99%)	1283 (30.47%)	1193 (28.33%)	809 (19.21%)	4211	169.27	**<0.001**	0.14	1.14 (1–1.3)	—
England Test	278 (12.31%)	823 (36.43%)	602 (26.65%)	556 (24.61%)	2259	289.43	**<0.001**	0.25	—	2.00 (1.7–2.4)
England T20 (Batters)	277 (35.79%)	153 (19.77%)	195 (25.19%)	149 (19.25%)	774	51.12	**<0.001**	0.18	1.86 (1.4–2.5)	—
England ODI (Batters)	687 (37.46%)	711 (38.77%)	329 (17.93%)	107 (5.83%)	1834	566.56	**<0.001**	0.39	6.41 (5–8.2)	—
England Test (Batters)	171 (13.51%)	652 (51.54%)	376 (29.72%)	66 (5.22%)	1265	663.81	**<0.001**	0.51	2.59 (1.9–3.6)	—
England T20 (Bowlers)	128 (17.51%)	199 (27.22%)	237 (32.42%)	167 (22.85%)	731	40.31	**<0.001**	0.17	—	—
England ODI (Bowlers)	225 (10.76%)	446 (22.28%)	804 (38.43%)	597 (28.54%)	2092	352.23	**<0.001**	0.29	—	2.66 (2.2–3.2)
England Test (Bowlers)	105 (11.18%)	160 (17.04%)	219 (23.32%)	455 (48.46%)	939	292.27	**<0.001**	0.39	—	4.34 (3.3–5.7)

Bold font denotes statistically significant chi-square at *p* < 0.05.

## Data Availability

Not applicable.
